# Targeting Next Generations to Change the Common Practice of Underpowered Research

**DOI:** 10.3389/fpsyg.2017.01184

**Published:** 2017-07-13

**Authors:** Rik Crutzen, Gjalt-Jorn Y. Peters

**Affiliations:** ^1^Department of Health Promotion, Care and Public Health Research Institute (CAPHRI), Maastricht University Maastricht, Netherlands; ^2^Faculty of Psychology and Education Science, Open University of the Netherlands Heerlen, Netherlands; ^3^Department of Work and Social Psychology, Faculty of Psychology and Neuroscience, Maastricht University Maastricht, Netherlands

**Keywords:** power, effect size, sample size, students, teaching, curriculum

Underpowered studies remain ubiquitous (Maxwell, 2004; Bakker et al., 2012; Button et al., 2013; Turner et al., 2013; Szucs and Ioannidis, 2017) despite strong pleas to change this practice (Cohen, 1988, 1990, 1992). As with any complex problem, multiple factors contribute to the ubiquity of conducting underpowered studies, and a wide range of efforts is needed to solve it. Most efforts to improve matters have focused on researchers and funding agencies. The present state of affairs evidences the unsuccessful education of both groups (e.g., Bakker et al., 2016). Therefore, in this contribution we propose to target next generations of researchers (i.e., students). We will briefly summarize the main reasons why underpowered research is a problem, discuss common defenses of underpowered studies, and then outline our proposed solutions.

The terms “power” and “underpowered” originally stem from the traditional null hypothesis significant testing (NHST) approach. However, there is consensus that basing conclusions on confidence intervals for effect size estimates is generally superior to relying on NHST (American Psychological Association, 2009). Hence, we will use a more general definition, where we redefine “underpowered” as “undersamplesized.” Sample size refers to number of data points for the variable or association of interest, not necessarily to the number of participants. This broader definition, therefore, also applies to non-NHST studies, such as studies aiming to obtain accurate parameter estimates. When we say “underpowered,” we mean “with too few data points” (not necessarily too few participants). Even when sticking to the narrower definition of power (e.g., not focusing accurate parameter estimates), then the estimated median power is 0.35 across studies in psychology (Bakker et al., 2012; Nuijten et al., 2015). In other words, conducting underpowered studies is a damaging yet regrettably common practice within psychology and other disciplines, such as neuroscience (Button et al., 2013).

## Why is it a problem to conduct underpowered studies?

Underpowered studies are problematic because they lead to biased conclusions (Maxwell, 2004; Christley, 2010; Turner et al., 2013; Kühberger et al., 2014). The reason behind these biased conclusions is that underpowered studies yield excessively wide sampling distributions for the sample estimates. This means that all parameters computed from the sample (e.g., effect sizes) can differ considerably from the population value, and also over replications. This partly explains why a large portion of the replications of 100 studies published in three psychology journals did not reproduce the original results (Open Science Collaboration, 2015; Peters and Crutzen, 2017). The biases due to underpowered studies are not limited to primary research, but can also distort meta-analytic evidence (Turner et al., 2013; Nuijten et al., 2015). For example, there is a replication paradox in the sense that pooling data from multiple studies can actually *decrease* accuracy of effect size estimates under publication bias (i.e., studies with results that are not statistically significant are less likely to be published, which decreases accuracy, Nuijten et al., 2015). Hence, it is better to conduct one large study (in terms of data points) than multiple smaller studies (Sijtsma, 2016). And when replicating a smaller study, it is warranted to assess the extent to which the replication results are consistent with an effect size large enough to have been detectable in the original study (Simonsohn, 2015).

These unpleasant methodological and statistical dynamics bear ethical implications. Besides the obvious undesirability of investing finite resources in producing what is likely to be misinformation, participants are also a finite and scarce resource. Using up this scarce resource for an underpowered study means that other (adequately powered) studies will have a harder time recruiting participants. In addition, for participants in a study, one incentive to participate may be the promise of contributing to scientific progress (Halpern et al., 2002). However, participating in underpowered studies might actually hamper scientific progress, as it leads to drawing wrong conclusions. Some situations are considered to provide dispensation from these methodological, statistical, and ethical concerns. We will discuss two examples.

## Situations where small samples are unjustifiably considered to be acceptable

A first example of such a situation is when studying a specific subgroup of the population (e.g., patients suffering from a rare disease), it can be very hard to recruit many participants. One could argue that in such cases some evidence is better than none (and some have, Edwards et al., 1997), but this line of reasoning is flawed. First, it implicitly assumes that power is exclusively a function of the number of participants. However, using intensive longitudinal methods (Naughton and Johnston, 2014; Inauen et al., 2016) or using better measurements (Peters et al., 2016) can yield sufficient power even if only a few participants are available. Second, it neglects the methodological and statistical dynamics outlined above, which mean that underpowered studies are often unable to contribute to in fact answer research questions. Note that often when researchers claim to study a rare population, they actually mean that the resources that they have at their disposal at that moment only allows collection of a limited sample (within a certain time frame or region). More resources often allow, for example, international coordination to collect data or collecting data over a longer time period. It is not the case that the interest that a researcher or organization has in answering a research question, or the urgency of obtaining that answer, void the methodological and statistical concerns outlined earlier. Sometimes, the more virtuous decision is to decide that current means do not allow studying the research question at hand. Moreover, the majority of studies in psychology uses student samples or other strata of the general population (Arnett, 2008). In all these cases, the argument that “there are not enough potential participants” is invalid.

Another example of a situation often presented as justifying small samples is when the study is a pilot study or early-phase trial (e.g., a median sample size of 76 participants; Arain et al., 2010). That is perfectly fine, if the aim of such studies is to identify unforeseen problems (Viechtbauer et al., 2015). However, an early-phase trial is not appropriate to get an accurate estimate of the effect size (Kraemer et al., 2006). This lack of accuracy affects future sample size calculations. For example, if researchers find an effect size of (Cohen's) *d* = 0.50 in an early-phase trial with *N* = 100, then the 95% confidence interval ranges from 0.12 to 0.91 (Maxwell et al., 2008).

These examples show that pleas for dispensation often do not hold up to close scrutiny. The methodological, statistical, and ethical concerns mean that in, almost all conceivable scenarios, and certainly those where researchers aim to answer a research question, sufficient power is required (or, more accurately, sufficient data points). While there are many reasons that the literature is rife with underpowered studies besides neglect to perform realistic power analyses [e.g., unanticipated difficulties with recruitment (Crutzen et al., 2014) and loss to follow-up (Crutzen et al., 2015), of which students should also be made aware], at the same time, psychological curricula promote continuous conducting of underpowered studies in a number of ways.

## A dysfunctional norm

First, evidence from underpowered studies is presented as sufficiently high-quality evidence to draw conclusions in textbooks of psychology. For example, the classic study on the bystander effect is cited in many textbooks of (social) psychology (Darley and Latané, 1968). In this study, the likelihood and speed of response (i.e., multiple outcomes) are compared between three groups that have sample sizes of respectively 13, 26, and 13 (Darley and Latané, 1968). Such small sample sizes provide very limited information as to how large an effect is in the population. This is not meant to critique this specific study that was conducted a long time ago, but to critique that it is still presented as such in current textbooks (e.g., Gleitman et al., 2011; p. 533). The same goes for examples in statistical textbooks that are often underpowered. Although examples using a few data points are very useful to show the mechanics of how a particular analytical method works (e.g., demonstrating calculations), no substantive conclusions should be drawn based on these examples, and they should be explicitly introduced as artificial examples.

Second, when students collect data (e.g., to fulfill requirements for their degree), they are often permitted to collect datasets lacking the power to draw conclusions. Resources (e.g., time and money) to collect data are often limited in such circumstances. This can lead to difficulties in collecting sufficient data for an adequately powered study. Underpowered research is often justified, and data collected nonetheless, with the argument that the aim is to teach students how to conduct research.

These practices disseminate a norm. The schema of a typical study that is taught is one with a sample consisting of dozens, rather than hundreds or thousands, of participants. Once students (future researchers) have adopted that implicitly communicated schema, this obstructs adoption of the message that hundreds of participants (or dozens of participants but many measurements) are required in a study where multiple effects are estimated and adequate power is desired. Students who learn for 4 years that it is feasible to learn about human psychology with a few dozen participants per study are likely to turn into researchers and policymakers who believe that it is feasible to learn about human psychology with a few dozen participants per study. Universities have the responsibility to disseminate norms that promote high-quality research, not the opposite.

## Targeting next generations

Taking this responsibility can take two forms. First, course materials should be updated. In the short run, brief supplemental materials can be added to the curriculum to make students aware of the strength of conclusions from studies with varying degrees of power. For example, visualizations of the sampling distributions of the relevant effect sizes can be shown (Peters and Crutzen, 2017). In the long run, textbooks should be updated so they more accurately reflect the current state of the art and critically discuss underpowered studies. The same can be done for textbooks in statistics and methodology. After all, one could argue that especially authors of statistics and methodology textbooks carry a heavy responsibility to set the right example. In fact, such textbooks are the obvious means to discuss the limitations of underpowered research more in detail.

The second venue is to target next generations by means of relatively small changes in the curricula of undergraduate and graduate degrees. This is a fairly simple structural change in comparison with, for example, changing funding policies (cf. Everett and Earp, 2015). In current curricula, students often have to conduct research in order to familiarize them with the research process of formulating a research question and setting up a study, collecting data, and, subsequently, analyzing this data, and drawing conclusions based on the results. We propose to separate these phases, so that instead of giving credit points for a thesis as a whole, these aspects are also graded separately (e.g., writing a research proposal, developing study materials, collecting data, analyzing data, and interpretation).

This would enable retaining the complete scientific cycle while eliminating underpowered studies. In such a situation, for those students who cannot feasibly collect data for an adequately powered study, many solutions exist. For example, students could design one study but analyse data from existing datasets (which becomes easier as full disclosure becomes commonplace, Crutzen et al., 2012; Peters et al., 2012, 2017; Wicherts, 2013). Students can also collect part of the data for a larger project, either within their universities or across multiple universities (e.g., the Collaborative Replications and Education Project CREP, 2013), which also gives students experience with collecting data. Another solution is to let students design a study, specify their exact design and analysis plan in a preregistration, and then simulating a dataset.

It is sometimes argued that a drawback of this approach is that students' intrinsic motivation decreases, as collecting and analyzing their own data might give them a sense of autonomy (reasoning in line with Ryan and Deci, 2000). Assuming that this is true, that decrease in intrinsic motivation does not automatically justify engaging in otherwise ethically circumspect research practices. Furthermore, encouraging students to think of another research question, one that is possible to study with the limited means available, teaches them a useful skill. Deciding on an interesting or even urgent research question does not entitle one to collect data to answer that research question regardless of practical obstacles that may preclude doing so in a methodologically and statistically acceptable manner.

## Conclusion

It is desirable and feasible to stop disseminating the dysfunctional norm of underpowered research. This would mean that participants, a scarce resource, are used efficiently and students actively contribute to conducting adequately powered studies. Moreover, the first experiences of students will be to collect data for adequately powered studies, which helps to set the norm as such. We believe that correcting what we teach students to reflect best practices (Finkel et al., 2015) can contribute to eliminating underpowered studies.

## Author contributions

Both authors developed the ideas presented in this manuscript. RC wrote the first draft of the manuscript, GP substantially contributed to the manuscript. Both authors agree on the final version of the manuscript.

### Conflict of interest statement

The authors declare that the research was conducted in the absence of any commercial or financial relationships that could be construed as a potential conflict of interest.
